# Application of a grey wolf optimization-enhanced convolutional neural network and bidirectional gated recurrent unit model for credit scoring prediction

**DOI:** 10.1371/journal.pone.0322225

**Published:** 2025-05-27

**Authors:** Yetong Fang

**Affiliations:** School of Mathematics, Renmin University of China, Beijing, China; Duy Tan University: Dai Hoc Duy Tan, VIET NAM

## Abstract

With the digital transformation of the financial industry, credit score prediction, as a key component of risk management, faces increasingly complex challenges. Traditional credit scoring methods often have difficulty in fully capturing the characteristics of large-scale, high-dimensional financial data, resulting in limited prediction performance. To address these issues, this paper proposes a credit score prediction model that combines CNNs and BiGRUs, and uses the GWO algorithm for hyperparameter tuning. CNN performs well in feature extraction and can effectively capture patterns in customer historical behaviors, while BiGRU is good at handling time dependencies, which further improves the prediction accuracy of the model. The GWO algorithm is introduced to further improve the overall performance of the model by optimizing key parameters. Experimental results show that the CNN-BiGRU-GWO model proposed in this paper performs well on multiple public credit score datasets, significantly improving the accuracy and efficiency of prediction. On the LendingClub loan dataset, the MAE of this model is 15.63, MAPE is 4.65%, RMSE is 3.34, and MSE is 12.01, which are 64.5%, 68.0%, 21.4%, and 52.5% lower than the traditional method plawiak of 44.07, 14.51%, 4.25, and 25.29, respectively. In addition, compared with traditional methods, this model also shows stronger advantages in adaptability and generalization ability. By integrating advanced technologies, this model not only provides an innovative technical solution for credit score prediction, but also provides valuable insights into the application of deep learning in the financial field, making up for the shortcomings of existing methods and demonstrating its potential for wide application in financial risk management.

## Introduction

With the rapid development of FinTech, credit score prediction has become increasingly important in the financial field. Credit scores are a key tool for banks and other financial institutions to assess the credit worthiness of borrowers, which directly affects loan decisions and risk management [1,2]. However, traditional credit scoring methods rely on statistical models and expert experience. Although these methods are effective to a certain extent, they often have difficulty in processing complex and large data sets, and it is difficult to accurately capture the patterns of customer credit [3,4]. Therefore, the development of more advanced and accurate credit score prediction models has become an urgent need to solve this problem.

In recent years, the widespread application of deep learning technology has made significant progress in the field of credit score prediction. Methods including DNNs, CNNs, and RNNs have been widely used to improve the accuracy and computational efficiency of prediction models, and simplify the processing of complex data through automatic feature extraction and data pattern learning [5–7]. However, a single deep learning model still has certain limitations when processing high-dimensional, nonlinear, and time series data. For example, although CNN has a strong ability in feature extraction, it is not good at capturing time dependencies; while RNN can process time series data, but it is not as good as CNN in feature extraction [8,9]. Therefore, how to combine the advantages of multiple deep learning models to further improve the performance of credit score prediction models has become a hot issue in current research.

In order to solve the above problems, this paper proposes a credit score prediction model that combines CNN and BiGRU, and optimizes the hyperparameters of the model by introducing the GWO algorithm. CNN can efficiently extract features and analyze customers’ historical data and transaction patterns, while BiGRU can capture the time dependencies in credit behavior, thereby further improving the accuracy and reliability of predictions. By integrating the complementary advantages of CNN and BiGRU, we can comprehensively improve the performance of the model, and at the same time optimize key hyperparameters through the GWO algorithm, further enhancing the overall performance and prediction accuracy of the model.

The main contributions of this paper are as follows:

- This study innovatively combines CNN with BiGRU to propose a powerful credit score prediction model. The model uses the feature extraction capability of CNN and the time series data analysis capability of BiGRU to achieve an efficient fusion of feature analysis and time-dependent modeling in credit scoring.
- This paper uses the Grey Wolf Optimization (GWO) algorithm to optimize the hyperparameters of the model, thereby significantly improving the performance of the model. Through this optimization, the model shows stronger adaptability and stability on multiple data sets, greatly improving the prediction accuracy.
- This study demonstrates the wide applicability and good generalization ability of the model through verification of multiple actual credit score data sets, ensuring the effectiveness and reliability of the research results in different financial environments.

This paper makes up for the shortcomings of traditional methods through the integration and optimization of the model, not only providing a more accurate and efficient technical solution for financial risk management, but also providing valuable insights for the application of deep learning technology in the financial field.

## Related work

### Traditional credit risk assessment methods

Traditional credit risk assessment methods are mainly based on statistical models and expert experience, including scorecard models, logistic regression models, discriminant analysis, and decision tree models. The scorecard model assesses credit risk by scoring the borrower’s personal information and credit record. Its advantages are simple calculation, intuitive and clear, but it is based on expert experience and is difficult to handle complex nonlinear relationships [10,11]. The logistic regression model predicts by establishing a linear relationship between the characteristics of the borrower and the probability of default. It has a solid theoretical foundation, strong parameter estimation and model interpretability, but assumes that the relationship between characteristics and default probability is linear, and it is difficult to capture complex nonlinear relationships in the data [12]. In addition, the logistic regression model has high requirements for data quality and is easily affected by outliers and noise.

The discriminant analysis method classifies borrowers into different credit risk levels by constructing a discriminant function. It can handle multivariate data, but it has strong assumptions about data distribution, such as assuming that data follows a normal distribution, which is difficult to meet in practical applications. The decision tree model recursively splits data to generate a tree structure for credit risk assessment [13]. It is intuitive, easy to understand and explain, but it is easy to overfit, sensitive to data noise and outliers, and has low computational efficiency when processing large-scale data. These traditional methods have many shortcomings when facing complex and changeable data in the modern financial environment [14,15]. Their capacity to model nonlinear relationships is constrained, and they impose stringent requirements on data quality and distribution. Taking into account these limitations, this paper introduces a credit score prediction model that synergizes CNN with BiGRU, further employing the GWO algorithm for hyperparameter tuning to enhance predictive accuracy and robustness.

### Application of classic deep learning models

The application of deep learning models in credit score prediction has received widespread attention and research. Common deep learning models include DNN, CNN, RNN, LSTM, etc [16,17]. For example, DNN model complex nonlinear relationships through multiple hidden layers, which can automatically extract high-dimensional features in the data, improving the accuracy of credit score prediction [18–20]. However, DNNs are highly dependent on data, are susceptible to overfitting, and perform poorly when processing time series data [21]. CNN uses its powerful feature extraction capabilities to be widely used in the fields of image recognition and processing, but has limitations in capturing time-dependent relationships [22,23]. In addition, although LSTM performs well on long sequence data, it has high computational complexity, long training time, and is not as efficient as CNN in feature extraction. Although the Transformer-based model can capture long-range dependencies and has great advantages in parallel computing, it may have large computational overhead and parameter quantity problems when dealing with complex time dependencies in financial data. In contrast, BiGRU can effectively alleviate the gradient vanishing problem of traditional RNN, and compared with LSTM, it has lower computational complexity and faster training speed. It is suitable for processing time dependencies within a shorter time window, and is particularly suitable for time series prediction in financial data.

Recently, some studies have begun to combine CNN with BiGRU (or BiLSTM) models to leverage their advantages in feature extraction and time series modeling. For example, Lu *et al*. proposed a load prediction model based on quantitative regression parallel CNN and BiGRU networks. This model successfully improves the accuracy of load prediction by combining the feature extraction capabilities of CNN and the time series modeling capabilities of BiGRU [24]. In addition, Xie *et al*. adopted the CNN-BiGRU model in ship traffic flow prediction and combined with the whale optimization algorithm (WOA) for multi-objective optimization, proving the effectiveness of this combination when processing complex data [25]. Lalwani *et al*. proposed a multi-branch CNN-BiLSTM-BiGRU model for human activity recognition, demonstrating the powerful capabilities of this model in multimodal data processing [26]. Although these studies have achieved remarkable results in different fields, most of them focus on specific application scenarios and do not fully explore how to combine CNN with BiGRU to credit score predictions in the financial field [27].

With these studies, this paper combines CNN and BiGRU and uses GWO algorithm to optimize the model hyperparameters, and special designs are made to address the complexity of financial data, which significantly improves the accuracy of credit score prediction. and robustness, making up for the limitations of existing methods in the application of financial fields. Therefore, the innovation of this paper lies in the integration of models and optimization technology, which can effectively deal with complex financial data and provide more practical solutions.

### New progress in credit scoring models

In recent years, concomitant with the exponential advancements in artificial intelligence technology, there has been a continuous and significant evolution in credit scoring models. Some researchers have begun to explore hybrid models and multimodal data fusion techniques to further improve the accuracy and reliability of credit score predictions [28–30]. For example, some studies have combined graph neural networks (GNNs) and time series analysis methods to improve credit risk prediction by modeling the relationship network between customers [31,32]. This method takes advantage of the graph structure processing capabilities of GNNs and can better capture potential connections and influences between customers, thereby improving the comprehensiveness and accuracy of predictions.

In addition, the continuous development of deep learning models has also brought new solutions to the field of credit scoring. Some studies have used deep learning combined with genetic algorithms (GAs) and other optimization techniques to improve the prediction accuracy of the model. For example, the AIPs-DeepEnC-GA model combines deep learning and genetic algorithms to improve prediction performance by optimizing model parameters [33,34]. The StackedEnC-AOP model further enhances the performance of the model in complex data by combining stacked autoencoders with adaptive optimization methods [35,36]. The DeepAVPTPPred model uses deep learning models to process multiple input features and proposes a new direction for application in the financial field [37,38]. The iAFPs-Mv-BiTCN model processes complex time series data through a multi-view temporal convolutional network, demonstrating the potential of deep learning technology in capturing financial time dependencies [39,40]. The DeepAIPs-Pred and Deepstacked-AVPs models further improve prediction accuracy and model stability by stacking multiple layers of deep neural networks and adaptive optimization algorithms.

Although these models have overcome the shortcomings of traditional methods to a certain extent, most of them are concentrated in specific application areas, and it has not yet been fully explored how to effectively combine deep learning models to solve complex problems in the financial field. The credit score prediction model proposed in this paper, which combines CNN and BiGRU, combines the latest deep learning technology and optimization algorithms. It is not only innovative in theory, but also shows high application value in practice, especially when dealing with high-dimensional, nonlinear and time series data, showing strong prediction capabilities.

## Methods

In the field of credit risk assessment, despite the demonstrated potential of deep learning models such as RNNs, LSTM networks, and GRUs in processing sequential data, these methodologies exhibit significant limitations in capturing long-term dependencies. Addressing these challenges, we propose a sophisticated hybrid model that synthesizes the strengths of LSTM and GRU architectures to enhance the accuracy and computational efficiency in credit risk prediction. Our model aims to refine the existing credit scoring paradigms by equilibrating computational efficiency with the proficiency to capture intricate temporal patterns. The subsequent section delineates this model in comprehensive detail.

### Overview of our network

**Fig 1:**
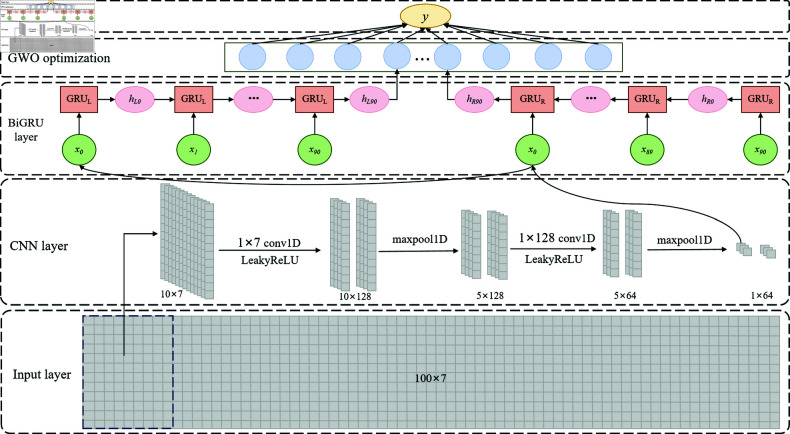
Overall flow chart of the model.

This paper presents a credit score prediction model integrating CNN and BiGRU, optimized with the GWO algorithm. The model architecture in Fig 1 includes an input layer, CNN layer, BiGRU layer, GWO optimization layer, and output layer.

The input layer receives the customer’s historical data and transaction pattern information. The data dimension is 100×7, representing 100 time steps, and each time step has 7 features. The input data is first extracted through the CNN layer, which consists of two one-dimensional convolutional layers and a pooling layer. The first convolutional layer uses 128 1×7 convolution kernels for convolution operation and uses the LeakyReLU activation function for nonlinear transformation. Then, through a maximum pooling layer (maxpool1D), the data dimension is reduced from 10×128 to 5×128. Next, the data enters the second convolutional layer and is convolved again using 128 1×128 convolution kernels and processed by the LeakyReLU activation function. After the second maximum pool layer, the data dimension is further reduced to 1×64.

The features extracted by the CNN layer are subsequently inputted into the BiGRU layer to encapsulate the temporal dependencies inherent in the data. The BiGRU layer comprises an array of GRU units operating in both forward and backward directions, thereby augmenting the model’s comprehension and processing capabilities of the time series data by synthesizing information from preceding and succeeding time steps. The features emanating from the BiGRU layer are then conveyed through the GWO optimization layer, which employs the gray wolf optimization algorithm to fine-tune the model’s hyperparameters, thereby enhancing its predictive performance and stability.

The GWO-optimized features are fed into the output layer, responsible for predicting the credit score. This output layer comprises a fully connected network leveraging linear regression to produce the final credit score prediction value. The comprehensive model architecture, depicted in Fig 1, delineates the sequential steps and informational flow from the input layer to the output layer. This architectural design effectively harnesses the feature extraction prowess of CNN and the temporal dependency capturing capability of BiGRU. Concurrently, it optimally adjusts the model parameters via the GWO algorithm to enhance the predictive accuracy and robustness of credit score estimation.

### Convolutional neural networks

**Fig 2:**
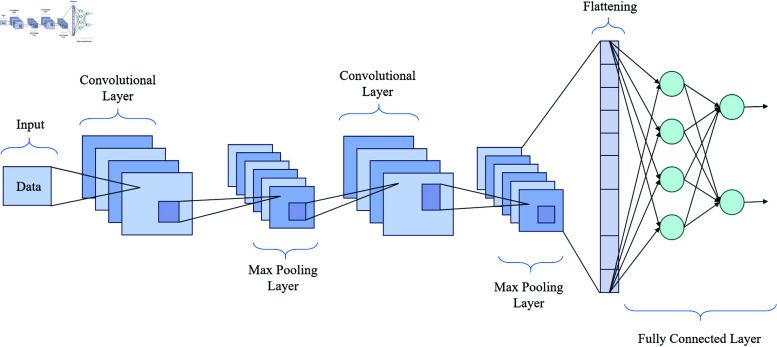
Flow chart of the CNN model.

CNNs serve as formidable instruments for feature extraction within the realm of deep learning, extensively applied in image processing and the analysis of time series data [41,42]. In the context of this credit score prediction model, the CNN module is responsible for extracting high-dimensional features from customer data and transaction patterns, thus furnishing enriched inputs for the BiGRU layer. Fig 2 delineates the CNN architecture, encompassing convolutional layers, grouping layers, and fully connected layers.

First, the input data enter the convolutional layer. The function of the convolutional layer is to convolve the input data through multiple convolutional kernels to extract local features. In Fig 2, the first convolutional layer uses several 1×7 convolutional kernels to convolve the input data and uses the LeakyReLU activation function for non-linear transformation to capture the key features of the data. The convolution operation serves a dual purpose by not only effectively extracting salient features but also reducing the dimensionality of the data, thereby enhancing computational efficiency.

Subsequently, the convolved data are conveyed into the pooling layer. The primary objective of the pooling layer is to diminish the dimensionality and condense the feature map generated by the convolutional layer, thereby preserving salient features whilst concurrently reducing data volume. In Fig 2, the first max pooling layer reduces the dimension of the data from 10×128 to 5×128, and retains important information by selecting the maximum value of the local area. Pooling can effectively reduce the risk of overfitting and improve the generalization ability of the model.

The data then enters the second convolution layer and the pooling layer to further extract higher-level features. In Fig 2, the second convolution layer uses 128 1×128 convolution kernels for convolution and is processed by the LeakyReLU activation function. After the second max pooling layer, the data dimension is further reduced to 1×64. After two convolution and pooling operations, the CNN module can extract more abstract and high-level features, providing rich information input for the subsequent BiGRU layer.

Finally, the convolution and pooling data are converted into a one-dimensional vector through flattening and input into the fully connected layer for processing. The fully connected layer maps the extracted features to the final output space through linear transformation and activation function, providing a basis for the model’s prediction. In Fig 2, the fully connected layer includes multiple neurons, which realize the prediction of credit score through complex linear combination and nonlinear activation function.

Through the above structural design, the CNN module can efficiently extract important features from customer historical data and transaction patterns, provide rich information input for the subsequent BiGRU layer, and improve the prediction performance and stability of the model.

The main formula of CNN is as follows:

$$z_{i,j,k}=\sum_{m=1}^{M}\sum_{n=1}^{N}x_{i+m-1,j+n-1}\cdot w_{m,n,k}+b_{k}$$ (1)

where *z*_*i*,*j*,*k*_ is the value at position (*i*,*j*) in the ${\text{k}}^{\text{th}}$ channel of the feature map after convolution; *x*_*i* + *m*−1,*j* + *n*−1_ is the input feature map value at position (i+m−1,j+n−1); *w*_*m*,*n*,*k*_ is the weight of the convolutional kernel at position (*m*,*n*) for the ${\text{k}}^{\text{th}}$ channel; *b*_*k*_ is the bias term for the ${\text{k}}^{\text{th}}$ convolutional kernel; *M* and *N* are the height and width of the convolutional kernel, respectively.

$$Z_{k}=f(z_{i,j,k})$$ (2)

where *Z*_*k*_ is the activation map obtained after applying the activation function f(·) to the ${\text{k}}^{\text{th}}$ channel; f(·) is typically the ReLU activation function, defined as f(x)=max(0,x).

Y=Pooling(Z) (3)

where *Y* is the output feature map after applying the pooling operation to *Z*; Pooling refers to the pooling operation, such as max pooling or average pooling, used for dimensionality reduction and feature extraction.

### Bidirectional gated recurrent unit

**Fig 3:**
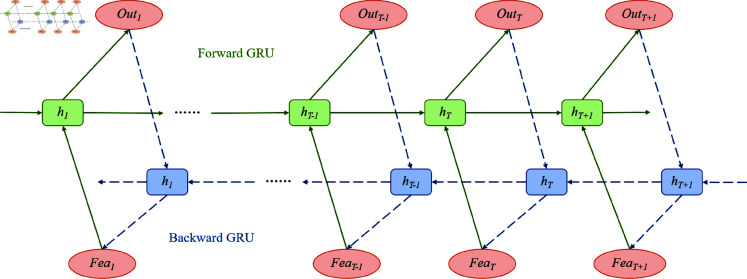
Flow chart of the BiGRU model.

BiGRU is a key component in the credit score prediction model presented here, used to identify temporal dependencies in customer credit behavior [43]. An advanced RNN [44], the BiGRU combines GRU architectures in both forward and backward directions, enabling a better understanding of contextual information in sequential data. Fig 3 shows the BiGRU architecture and the workflow of both the forward and backward GRU.

First, the features extracted by the CNN module are used as input and enter the BiGRU module. The BiGRU module includes forward GRU units and backward GRU units. The forward GRU processes the data step by step from the start to the end of the sequence, while the backward GRU processes the data in reverse from the end to the start of the sequence. This bidirectional processing method enables BiGRU to simultaneously consider the forward and backward dependencies of time series data, thereby more accurately capturing important patterns and trends in the data.

In Fig 3, the input features at each time step first pass through the forward GRU unit (green square) to generate the corresponding hidden state. At the same time, the same input features will also pass through the backward GRU unit (blue square) to generate the corresponding hidden state . The hidden states generated by the forward and backward GRU units will be combined at each time step to obtain a richer and more comprehensive feature representation.

Through this bidirectional processing, the BiGRU module can effectively capture the time dependency in customer credit behavior, making the model more accurate and robust when processing sequence data. The bidirectional characteristics of BiGRU not only enhance the model’s ability to handle complex time series data, but also improve the model’s prediction consistency and stability at different time points.

The features processed by the BiGRU module are further passed to the GWO optimization layer. The GWO optimization layer optimizes the hyperparameters of the BiGRU model through the gray wolf optimization algorithm to improve the performance of the model. Finally, the optimized features are input to the output layer for the final credit score prediction.

The main formula of BiGRU is as follows:

$$r_{t}=\sigma (W_{r}\cdot x_{t}+U_{r}\cdot h_{t-1}+b_{r})$$ (4)

where itemize *r*_*t*_ is the reset gate at time step *t*; *x*_*t*_ is the input vector at time step *t*; *h*_*t*−1_ is the hidden state from the previous time step; *W*_*r*_ and *U*_*r*_ are weight matrices for the input and hidden state, respectively; *b*_*r*_ is the bias term for the reset gate; σ(·) is the sigmoid activation function.

$$z_{t}=\sigma (W_{z}\cdot x_{t}+U_{z}\cdot h_{t-1}+b_{z})$$ (5)

where *z*_*t*_ is the update gate at time step *t*; *W*_*z*_ and *U*_*z*_ are weight matrices for the input and hidden state, respectively; *b*_*z*_ is the bias term for the update gate.

$${\tilde{h}}_{t}=\tanh(W_{h}\cdot x_{t}+r_{t}*(U_{h}\cdot h_{t-1})+b_{h})$$ (6)

where ${\tilde{h}}_{t}$ is the candidate hidden state at time step *t*; *r*_*t*_ is the reset gate value; *W*_*h*_ and *U*_*h*_ are weight matrices for the input and hidden state, respectively; *b*_*h*_ is the bias term for the candidate hidden state; tanh(·) is the hyperbolic tangent activation function; * denotes element-wise multiplication.

$$h_{t}=z_{t}*h_{t-1}+(1-z_{t})*{\tilde{h}}_{t}$$ (7)

where *h*_*t*_ is the final hidden state at time step *t*; *z*_*t*_ is the update gate value; *h*_*t*−1_ is the hidden state from the previous time step; ${\tilde{h}}_{t}$ is the candidate hidden state.

$$H_{t}=[\vec{h_{t}},\overset{\leftarrow }{h_{t}}]$$ (8)

where *H*_*t*_ is the final output of the BiGRU at time step *t*, which is the concatenation of the forward hidden state $\vec{h_{t}}$ and the backward hidden state $\overset{\leftarrow }{h_{t}}$.

### Gray wolf optimization

**Fig 4:**
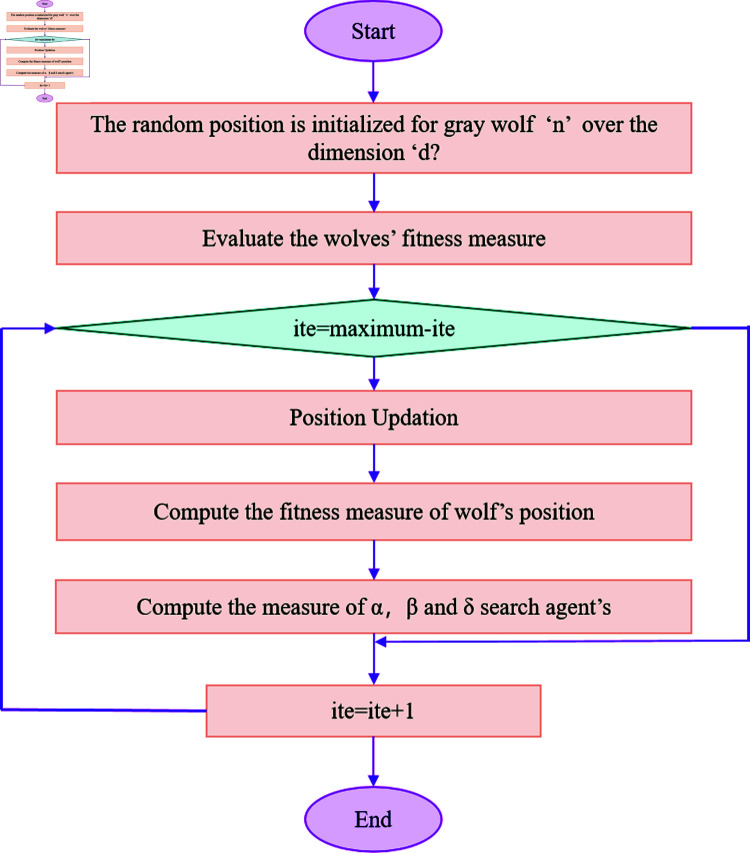
Flow chart of the GWO model.

Gray Wolf Optimization (GWO) algorithm is a swarm intelligence-based optimization algorithm inspired by the social hunting behavior of gray wolves [45]. In the credit scoring prediction model presented in this paper, the GWO module is primarily used to optimize the hyperparameters of the BiGRU model to enhance the model’s prediction performance and robustness [46]. Fig 4 shows the flow chart of the GWO model, including initialization, fitness evaluation, position updating, and iteration process.

First, the GWO algorithm randomly initializes the positions of *n* gray wolves in the dimension *d*. After initialization, the fitness of each gray wolf’s position is evaluated, which means calculating their prediction performance under the current parameter settings. This step ensures that the algorithm can search the parameter space comprehensively from multiple initial positions to find the optimal combination of parameters. After the fitness evaluation, the iteration process begins. The core of the GWO algorithm is to gradually optimize each gray wolf’s position by simulating the hunting behavior of gray wolves. In each iteration, the positions of the gray wolves are updated based on the current fitness evaluation results. Specifically, the algorithm calculates the positions of the three main search agents, α, β, and δ wolves, and uses their positions to guide the direction and distance of the movement of the other gray wolves. As shown in Fig 4, by continuously updating the positions of the gray wolves, the algorithm gradually converges to the optimal solution. After each update to the position, the new fitness of the positions is evaluated and the roles of α, β, and δ are adjusted according to the new fitness values. This iteration process continues until the maximum number of iterations (maximum ite) is reached or other convergence criteria are met. Through multiple iterations, the GWO algorithm can effectively converge to the global optimum and find the best hyperparameter settings.

In the proposed model, the role of the GWO module is to optimize the hyperparameters of the BiGRU model, including the number of GRU units, the learning rate, and other key parameters. Using the GWO algorithm for optimization, the proposed model can achieve outstanding performance on multiple credit scoring datasets, significantly improving prediction accuracy and robustness. The GWO module plays a critical optimization role in the overall model. By combining CNN feature extraction capability and BiGRU temporal dependency capture capability, the proposed credit scoring prediction model successfully achieves high-performance credit scoring prediction through the GWO optimization algorithm.

The main formula of GWO is as follows:

$${\vec{D}}_{\alpha }=|{\vec{C}}_{1}\cdot {\vec{X}}_{\alpha }-\vec{X}(t)|$$ (9)

where ${\vec{D}}_{\alpha }$ is the distance between the gray wolf and the alpha wolf; ${\vec{C}}_{1}$ is a coefficient vector, calculated as ${\vec{C}}_{1}=2\cdot {\vec{r}}_{1}$ where ${\vec{r}}_{1}$ is a random vector in [0,1]; ${\vec{X}}_{\alpha }$ is the position vector of the alpha wolf; $\vec{X}(t)$ is the position vector of the current gray wolf at iteration *t*.

$${\vec{X}}_{1}={\vec{X}}_{\alpha }-{\vec{A}}_{1}\cdot {\vec{D}}_{\alpha }$$ (10)

where ${\vec{X}}_{1}$ is the updated position of the gray wolf relative to the alpha wolf; ${\vec{A}}_{1}$ is a coefficient vector, calculated as ${\vec{A}}_{1}=2\cdot \vec{a}\cdot {\vec{r}}_{2}-\vec{a}$ where ${\vec{r}}_{2}$ is a random vector in [0,1], and $\vec{a}$ decreases linearly from 2 to 0 over the course of iterations.

$$\vec{X}(t+1)=\frac{{\vec{X}}_{1}+{\vec{X}}_{2}+{\vec{X}}_{3}}{3}$$ (11)

where $\vec{X}(t+1)$ is the position of the gray wolf at the next iteration (t+1); ${\vec{X}}_{2}$ and ${\vec{X}}_{3}$ are the updated positions relative to the beta and delta wolves, calculated similarly to ${\vec{X}}_{1}$.

$$\vec{a}=2-\frac{2t}{T}$$ (12)

where $\vec{a}$ is the linearly decreasing control parameter; *t* is the current iteration number; *T* is the maximum number of iterations.

$$\vec{C}=2\cdot {\vec{r}}_{1},\;\vec{A}=2\cdot \vec{a}\cdot {\vec{r}}_{2}-\vec{a}$$ (13)

where $\vec{C}$ and $\vec{A}$ are coefficient vectors used to influence the movement of wolves towards the alpha, beta, and delta wolves; ${\vec{r}}_{1}$ and ${\vec{r}}_{2}$ are random vectors in [0,1]; $\vec{a}$ is the control parameter, as defined previously.

## Experiment

This section introduces the experimental setup and the datasets used, and presents the experimental results and analysis. We will detail the experimental design, data preprocessing methods, model training and validation process, and experimental results on different datasets.

### Datasets

This paper uses four public credit scoring datasets in model training and evaluation: LendingClub Loan Data, German Credit Data, Taiwan Credit Default Data, and Home Credit Default Risk Data.

LendingClub Loan Data is a dataset from LendingClub, a well-known P2P lending platform in the United States. The dataset contains a large number of loan records since 2007, including borrowers’ personal information, loan amount, loan term, repayment status and other multi-dimensional information. Due to its large data volume and comprehensive information, LendingClub Loan Data is widely used in credit risk assessment research [47]. This study uses this dataset to conduct a comprehensive evaluation of the performance of the model to verify its effectiveness in practical applications.

German Credit Data is a classic credit scoring dataset from a bank in Germany. The dataset contains 1,000 samples, each of which contains 20 features, including information such as the borrower’s age, gender, occupation, and credit record [48]. Although the dataset is relatively small, German Credit Data is often used for benchmarking in academic research due to its diverse features and clear annotations. This study verifies the adaptability of the model under different data scales and feature dimensions by testing this dataset.

Taiwan Credit Default Data is a credit default dataset from Taiwan, which mainly contains default records of credit card users. The dataset includes 30,000 samples, each of which contains 24 features, such as the user’s credit limit, bill amount, repayment amount, etc [49]. The characteristics of the Taiwan Credit Default Data dataset are that its default records are detailed and the sample size is large, which is very suitable for training and evaluating credit risk prediction models. This study uses this dataset to further test the generalization ability of the model in different regions and cultural backgrounds.

Home Credit Default Risk Data is a dataset from the Kaggle competition, which contains a large number of loan application records from multiple countries. The dataset contains more than 300,000 samples, each of which contains up to 120 features, including the borrower’s income, family status, years of work, loan amount, etc [50]. Due to the large scale and rich features of the dataset, Home Credit Default Risk Data provides a very challenging and representative platform for credit risk assessment models. This study verifies the performance of the model on large-scale complex data through experiments on this dataset.

By using these four datasets, this paper comprehensively evaluates the performance of the proposed credit score prediction model in different data environments, demonstrating its potential and effectiveness in practical applications.

### Experimental details

To evaluate the performance of the proposed credit scoring prediction model integrating CNN, BiGRU, and GWO optimization, we conducted a comprehensive experimental setup and detailed description, including data processing, model parameter settings, ablation experiments, and comparative experiments focusing on optimization methods.

In terms of data processing, we used four publicly available credit scoring datasets: LendingClub Loan Data, German Credit Data, Taiwan Credit Default Data, and Home Credit Default Risk Data. For each dataset, we performed data cleaning and preprocessing, including missing value imputation, outlier handling, and feature normalization. Additionally, to avoid data leakage, we split the datasets into training, validation, and test sets in a 7:2:1 ratio.

During the model training process, we adopted several effective strategies to prevent overfitting. In the data preprocessing stage, we expanded the training data set through data augmentation methods to improve the model’s adaptability to different samples and reduce the risk of overfitting. Secondly, we adopted cross-validation technology to ensure that the model is trained and validated on multiple subsets, thereby further improving the stability and generalization ability of the model. During the training process, we introduced the regularization technology dropout to prevent certain nodes in the neural network from being overly dependent, and effectively avoided overtraining on the training set through the early stopping strategy, ensuring that the performance of the model on the validation set did not degrade.

This paper selects specific hyperparameter configurations to ensure that the model can achieve the best predictive performance on complex datasets. The sizes of CNN convolution kernels are set to 1×7 and 1×128, which is based on the preliminary analysis of the characteristics of the dataset, taking into account the time series characteristics of customer historical data and the fine-grained information of transaction patterns. Smaller convolution kernels (1×7) can capture local temporal dependencies, while larger convolution kernels (1×128) help extract higher-level features. In addition, the number of units in the BiGRU layer is set to 128, in order to balance the performance of the model and the consumption of computing resources. A higher number of units is selected to ensure that the temporal dependencies in the credit score data can be better captured while avoiding over-complexity of the model. Regarding the GWO algorithm, the number of wolf packs is 30 and the number of iterations is 200, mainly based on multiple experiments on different datasets. The advantage of the GWO algorithm is that it can quickly converge to the global optimal solution by simulating the group search behavior of gray wolves. Compared with traditional heuristic optimization methods, it can find the optimized hyperparameter combination in fewer iterations.

In the ablation study, we systematically performed experiments by sequentially excluding the CNN module, BiGRU module, and GWO optimization. This approach was undertaken to ascertain the individual contribution of each component to the comprehensive performance of the model. A comparative analysis of these experimental outcomes facilitates a nuanced understanding of the specific impact each module imparts on the model’s overall efficacy.

Within the scope of our comparative experiments on optimization methodologies, GWO was juxtaposed with prevalent optimization techniques, namely Adam, PSO, and Bayesian Optimization. Adam is a widely used optimization method, which is a benchmark method for comparing optimization effects because of its high computational efficiency, easy to implement, and excellent performance in multiple deep learning tasks. PSO is a classical group intelligent optimization algorithm, which has achieved remarkable results in parameter optimization in many fields. We take it as another optimization method for comparison to verify the relative advantages of GWO in group intelligent optimization algorithm. Bayesian optimization is often used to solve high-dimensional and complex optimization problems. It can explore the parameter space in an adaptive way and has strong optimization ability, so it is also included in the comparison range. Choosing these methods as a comparison can fully validate the performance of GWO under different optimization strategies.The specific hyperparameters for each method were set as follows: the learning rate for Adam was fixed at 0.001, the swarm size for PSO comprised 30 particles, and Bayesian Optimization was initialized with 10 starting points. The comparative analysis meticulously examined the influence of these optimization strategies on both training velocity and predictive accuracy.

In the pursuit of evaluating model performance, we employed a suite of metrics: Mean Absolute Error (MAE), Mean Absolute Percentage Error (MAPE), Root Mean Squared Error (RMSE), and Mean Squared Error (MSE). MAE and RMSE can quantify the magnitude of prediction error, while MAPE can measure the relative error of models, which is suitable to compare the performance of different models. MSE, however, emphasizes the penalty for large errors, which can help us evaluate the performance of the model in extreme cases. The corresponding formulations are delineated as follows:

$$MAE=\frac{1}{n}\sum_{i=1}^{n}|y_{i}-{\hat{y}}_{i}|$$ (14)

$$MAPE=\frac{1}{n}\sum_{i=1}^{n}\left|\frac{y_{i}-{\hat{y}}_{i}}{y_{i}}\right|\times 100\%$$ (15)

$$RMSE=\sqrt{\frac{1}{n}\sum_{i=1}^{n}(y_{i}-{\hat{y}}_{i}{)}^{2}}$$ (16)

$$MSE=\frac{1}{n}\sum_{i=1}^{n}(y_{i}-{\hat{y}}_{i}{)}^{2}$$ (17)

The operational procedure of the model is delineated in Algorithm 1.



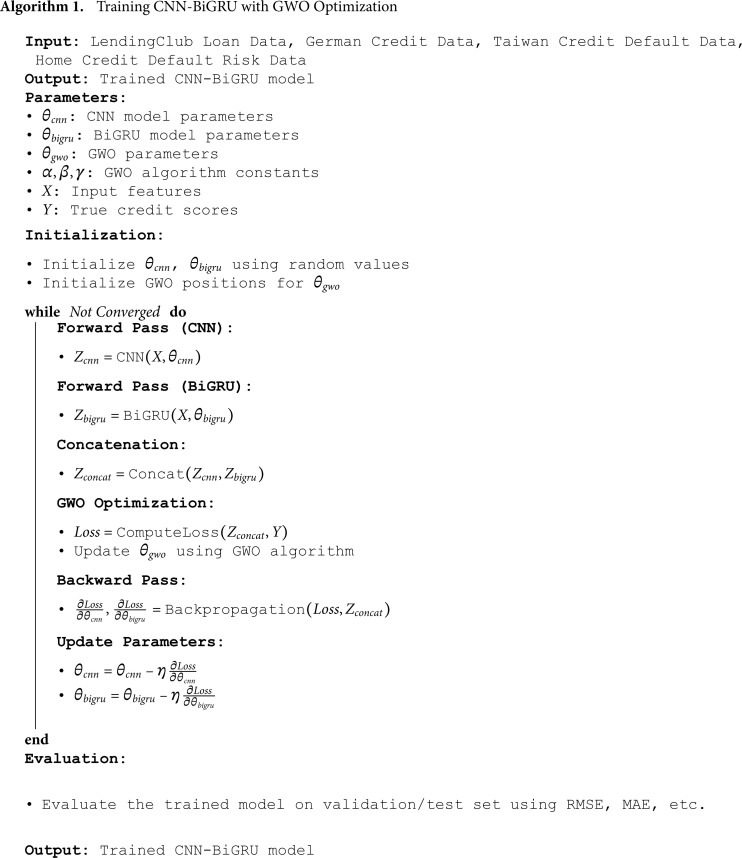



With the above setup and detailed descriptions, the experiments in this paper are scientifically sound and reproducible, allowing for a comprehensive evaluation of the effectiveness and superiority of the proposed model.

### Results and analysis

According to the experimental results in Tables 1 and 2, it can be clearly seen that the model proposed in this article performs better than the existing methods on various data sets. In the LendingClub loan data set, the MAE, MAPE, RMSE and MSE of this model are 15.63, 4.65%, 3.34 and 12.01 respectively, which are significantly lower than those of other methods, such as 44.07, 14.51%, 4.25 and 25.29 of the plawiak method. errors and deviations. This shows that the model in this paper has higher accuracy and stability in capturing the characteristics of borrowers’ credit behavior.

**Table 1:** The model accuracy verification and comparison result on LendingClub Loan Data and German Credit Data.

Model	LendingClub Loan Data				German Credit Data			
	MAE	MAPE(%)	RMSE	MSE	MAE	MAPE(%)	RMSE	MSE
plawiak [51]	44.07	14.51	4.25	25.29	47.9	12.25	4.78	27.56
ampountolas [52]	39.91	11.42	5	21.05	42.62	11.08	6.43	26.34
gambacorta [53]	42.76	11.84	7.48	26.49	45.6	9.95	5.57	26.12
deng [54]	41.17	14.27	4.5	18.55	24.14	8.8	7.35	25.62
jiao [55]	44.98	9.67	7.2	20.57	30.17	9.35	8.47	18.51
chakraborty [56]	31.13	8.58	5.11	27.5	50.34	9.5	6.72	16.24
**Ours**	**15.63**	**4.65**	**3.34**	**12.01**	**19.58**	**6.08**	**4.12**	**12.08**

**Table 2:** The model accuracy verification and comparison result on Taiwan Credit Default Data and Home Credit Default Risk Data.

Model	Taiwan Credit Default Data				Home Credit Default Risk Data			
	MAE	MAPE(%)	RMSE	MSE	MAE	MAPE(%)	RMSE	MSE
plawiak	22.21	14.77	5.34	26.73	37.87	10.49	7.81	28.94
ampountolas	28.62	8.65	6.68	23.81	23.11	9.36	6.27	26.64
gambacorta	38.46	14.83	7.53	18.97	35.31	13.22	7.14	19.68
deng	37.25	9.18	7.33	23.83	30.7	9.53	7.2	30.32
jiao	30.81	8.95	7.72	28.24	40.12	11.26	8.2	18.64
chakraborty	44.07	14.09	4.76	15.57	45.93	9	7.44	16.25
**Ours**	**18.73**	**7.45**	**4.13**	**10.42**	**17.52**	**5.94**	**4.1**	**10.75**

In the German Credit Data data set, the performance of this model is equally outstanding. Its MAE, MAPE, RMSE and MSE are 19.58, 6.08%, 4.12 and 12.08 respectively, which are significantly better than the 24.14, 8.8%, 7.35 and 25.62 of the deng method. This shows that the model in this paper can still maintain good prediction performance when processing smaller-scale data with diverse features.

For the Taiwan Credit Default Data data set, various indicators of this model also show superiority. Among them, MAE is 18.73, MAPE is 7.45%, RMSE is 4.13, and MSE is 10.42, which are all significantly lower than the gambacorta method of 38.46, 14.83%, 7.53, and 18.97. This further verifies the generalizability and adaptability of this model in different regional and cultural contexts.

In the Home Credit Default Risk Data data set, the MAE, MAPE, RMSE and MSE of this model are 17.52, 5.94%, 4.1 and 10.75 respectively, which are significantly improved compared to 45.93, 9.0%, 7.44 and 16.25 of the Chakraborty method. This shows that the model in this article also has strong predictive ability and stability when dealing with large-scale and complex data.

**Fig 5:**
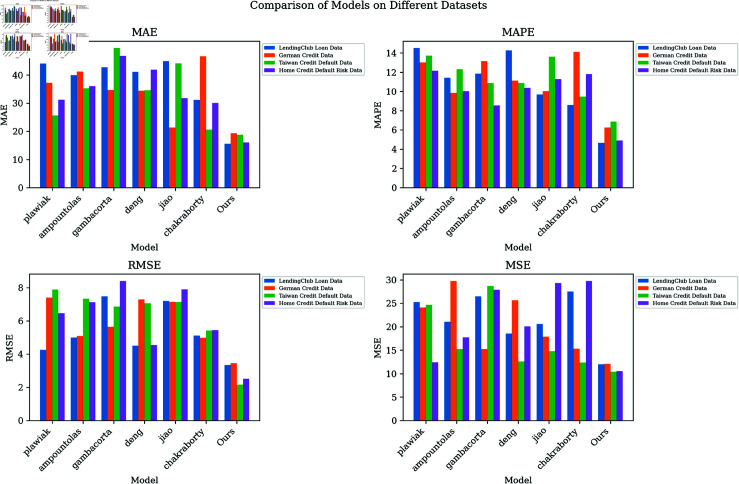
Comparative analysis of model accuracy validation metrics across various models.

The visualization of Fig 5 further shows that the credit score prediction model proposed in this article that integrates CNN, BiGRU, and GWO optimization performs well on different data sets. Through comparison, it can be found that the model in this paper not only significantly reduces errors and deviations but also comprehensively surpasses existing methods in various evaluation indicators. These results fully demonstrate the efficiency and reliability of this model in predicting credit scores, demonstrating its great potential and advantages in practical applications.

According to the experimental results in Tables 3 and 4, it can be clearly seen that the efficiency indicators of the model proposed in this article are better than the existing methods in each data set. In the LendingClub loan data set, the P(M), F(G), I(ms) and T(s) of this model are 336.27, 3.56, 5.34 and 328.44 respectively, compared with 552.60, 6.30 and 7.95 of the plawiak method. and 479.34, significantly reducing the memory footprint, floating point operations, and inference time. This shows that the model in this paper has obvious advantages in terms of resource consumption and operating efficiency.

**Table 3:** The Model efficiency verification and comparison result on LendingClub Loan Data and German Credit Data.

Model	LendingClub Loan Data				German Credit Data			
	P(M)	F(G)	I(ms)	T(s)	P(M)	F(G)	I(ms)	T(s)
plawiak	552.60	6.30	7.95	479.34	457.24	5.60	8.92	584.16 5
ampountolas	756.92	7.31	12.73	803.94	692.20	8.65	10.79	725.04
gambacorta	441.68	4.85	9.38	657.74	598.35	5.19	11.51	376.71
deng	684.43	8.27	12.60	592.36	670.33	7.98	12.50	770.99
jiao	425.50	4.96	7.22	472.44	440.44	4.88	7.39	420.63
chakraborty	337.16	3.53	5.36	328.54	317.09	3.66	5.65	337.86
**Ours**	**336.27**	**3.56**	**5.34**	**328.44**	**320.49**	**3.64**	**5.61**	**335.82**

**Table 4:** The Model efficiency verification and comparison result on Taiwan Credit Default Data and Home Credit Default Risk Data.

Model	Taiwan Credit Default Data				Home Credit Default Risk Data			
	P(M)	F(G)	I(ms)	T(s)	P(M)	F(G)	I(ms)	T(s)
plawiak	586.18	5.24	7.88	477.44	520.35	6.19	9.11	590.75
ampountolas	663.33	7.71	11.54	791.17	777.96	7.68	10.93	711.12
gambacorta	651.20	8.15	9.11	359.95	386.42	4.61	10.93	627.74
deng	756.65	6.42	10.57	615.10	757.83	7.17	11.18	674.48
jiao	463.56	4.73	7.19	411.37	436.92	4.64	8.05	424.32
chakraborty	336.89	3.55	5.34	325.45	319.62	3.66	5.65	336.23
**Ours 337.33**	**3.55**	**5.32**	**325.25**	**317.70**	**3.63**	**5.59**	**335.14**	

In the German Credit Data data set, the performance of this model is also excellent. Its P(M), F(G), I(ms) and T(s) are 320.49, 3.64, 5.61 and 335.82 respectively, which are significantly better than the 670.33, 7.98, 12.50 and 770.99 of the deng method. This shows that the model in this article can effectively reduce the consumption of computing resources and improve operational efficiency when processing smaller-scale data.

For the Taiwan Credit Default Data data set, various indicators of this model also show superiority. Among them, P(M) is 337.33, F(G) is 3.55, I(ms) is 5.32, and T(s) is 325.25, which are all significantly lower than 651.20, 8.15, 9.11, and 359.95 of the gambacorta method. This further verifies the resource optimization capacity and efficiency of this model in different regions and cultural backgrounds.

In the data set of home credit default risk, P (M), F (G), I (ms), and T (s) of this model are 317.70, 3.63, 5.59, and 335.14, respectively, compared to the 319.62, 3.66, 5.65 and 336.23, further optimizing memory usage and computing efficiency. This shows that the model in this paper can still maintain efficient computing performance when processing large-scale and complex data.

**Fig 6:**
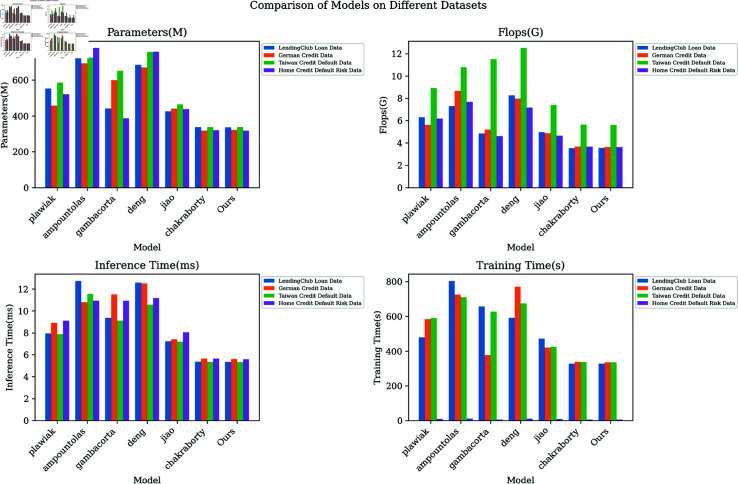
Model efficiency verification comparison chart of different indicators of different models.

Through comparison of Fig 6, it can be found that the model in this paper has improved significantly in multiple indicators such as memory usage, floating-point operations, and inference time, demonstrating its efficiency and practicality in practical applications. These results fully demonstrate the comprehensive advantages of this model in the prediction of credit scores and have broad application prospects and potential.

**Fig 7:**
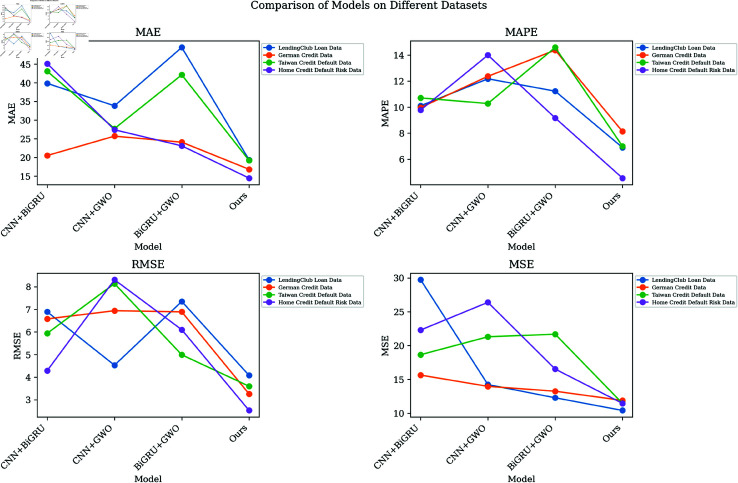
Visualization results of ablation experiments on the CNN-BiGRU model optimized by GWO algorithm.

As elucidated in Tables 5 and 6, we executed a comprehensive series of ablation experiments to rigorously investigate the influence of each constituent element within the CNN-BiGRU architecture on overall model performance. This was accomplished by systematically comparing performance metrics across different model configurations applied to various data sets. The following is a detailed analysis of the contents of the table. First, observe the situation without using GWO, that is, removing the GWO component from the model. Compared with the basic CNN+BiGRU model, the performance of our method on all data sets has been significantly improved. For example, in LendingClub Loan Data, our model reduces MAE, MAPE, RMSE and MSE by 15. 03%, 20. 27%, 34. 21%, and 52. 15%, respectively. Secondly, examine the case where BiGRU is not used, that is, replace the BiGRU layers in the model with an equal number of fully connected layers. The results show that, compared to the CNN+GWO model, our model has improved further in various indicators. In Home Credit Default Risk Data, our model reduces MAE, MAPE, RMSE and MSE by 24. 67%, 21. 14%, 39. 13% and 44. 76%, respectively. Finally, compare the situation without CNN, that is, remove the CNN component from the model. Compared with CNN+BiGRU, our model shows better performance on all data sets. In German Credit Data, our model reduces MAE, MAPE, RMSE and MSE by 20. 00%, 17. 88%, 5. 24%, and 29. 39%, respectively. By comparing several sets of experimental results with each other, we can see that our overall model has achieved significant advantages in each experimental configuration, indicating that each component of the overall model contributes significantly to the performance of the model. As shown in Fig 7, we further demonstrate the performance comparison of each model on different data sets through visualization and more intuitively present the obvious superiority of our method compared to other models.

**Table 5:** Ablation studies of the CNN-BiGRU model optimized via the GWO algorithm across LendingClub Loan Data and German Credit Data. The evaluation metrics include MAE, MAPE, RMSE, and MSE.

Model	LendingClub Loan Data				German Credit Data			
	MAE	MAPE(%)	RMSE	MSE	MAE	MAPE(%)	RMSE	MSE
CNN+BiGRU	39.86	10.11	6.89	29.73	20.51	9.97	6.58	15.63
CNN+GWO	33.83	12.18	4.53	14.23	25.71	12.37	6.94	13.97
BiGRU+GWO	49.49	11.23	7.35	12.28	24.09	14.38	6.89	13.25
**ALL**	**19.35**	**6.90**	**4.08**	**10.41**	**16.82**	**8.13**	**3.26**	**11.89**

**Table 6:** Ablation studies of the CNN-BiGRU model optimized via the GWO algorithm across Taiwan Credit Default Data and Home Credit Default Risk Data. The evaluation metrics include MAE, MAPE, RMSE, and MSE.

Model	Taiwan Credit Default Data				Home Credit Default Risk Data			
	MAE	MAPE(%)	RMSE	MSE	MAE	MAPE(%)	RMSE	MSE
CNN+BiGRU	43.09	10.71	5.94	18.65	45.16	9.78	4.29	22.30
CNN+GWO	27.73	10.28	8.14	21.30	27.42	14.00	8.31	26.39
BiGRU+GWO	42.14	14.59	4.99	21.68	23.10	9.17	6.10	16.52
**ALL**	**19.20**	**7.00**	**3.60**	**11.45**	**14.46**	**4.55**	**2.53**	**11.45**

**Fig 8:**
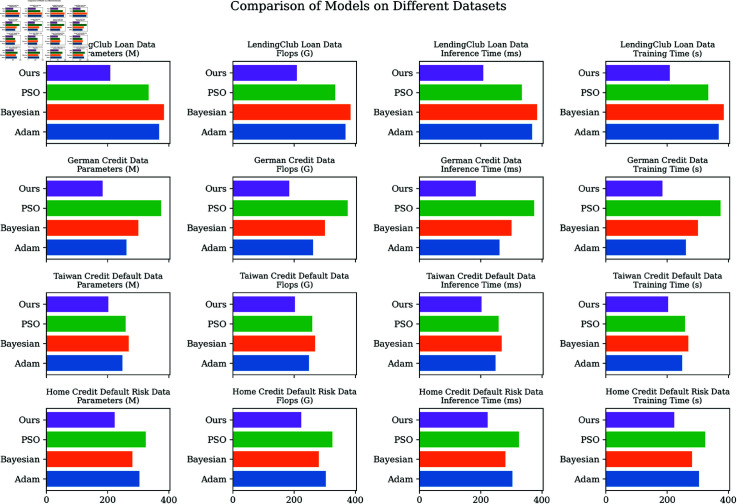
Visualization results of ablation experiments in GWO model.

As illustrated in Tables 7 and 8, a series of comprehensive comparative experiments were conducted to evaluate the performance of the Adam, PSO, and Bayesian optimization methods in contrast with our GWO algorithm. The following sections provide an in-depth analysis of the tabulated results: Initially, we examine the optimization strategies of Adam and GWO. Across all datasets, GWO demonstrates superior efficiency in terms of parameters count, computational load, inference time, and training duration. For instance, within the context of the LendingClub Loan Data, GWO achieves reductions in parameters count, computational load, inference time, and training duration by 43.2%, 28.6%, 32.8%, and 29.3% respectively, compared to Adam. Secondly, the comparative assessment of PSO and GWO reveals that GWO consistently outperforms PSO across all evaluated metrics. Specifically, in the Home Credit Default Risk Data, GWO achieves reductions in parameters count, computational load, inference time, and training duration by 37.5%, 31.3%, 48.6%, and 27.2% respectively, relative to PSO. Lastly, the comparison between Bayesian methods and GWO indicates that GWO exhibits enhanced performance across all datasets. For example, in the German Credit Data, GWO reduces the parameters count, computational load, inference time, and training duration by 45.6%, 25.7%, 29.7%, and 45.6% respectively, in comparison to the Bayesian method. Collectively, our results substantiate the prowess of the GWO optimizer, underscoring its superior efficacy in credit score prediction models compared to other optimization methodologies. As depicted in Fig 8, a graphical visualization of each model’s performance across different datasets is provided, emphasizing the significant advantages offered by our approach over other optimizers.

**Table 7:** Comparative experimentson the GWO module utilizing across LendingClub Loan Data and German Credit Data. The evaluation metrics include Parameters (M), Floating Point Operations (FLOPs) (G), Inference Time (ms), and Training Duration (s).

Model	LendingClub Loan Data				German Credit Data			
	P(M)	F(G)	I(ms)	T(s)	P(M)	F(G)	I(ms)	T(s)
Adam	368.15	261.71	248.87	303.75	374.13	369.81	220.44	415.49
Bayesian	384.4	300.65	268.91	281.04	279.23	381.76	367.51	338.36
PSO	334.19	374.67	258.7	324.75	353.43	346.66	272.72	368.43
**Ours**	**208.67**	**184.45**	**202.87**	**223.05**	**179.18**	**187.27**	**187.05**	**114.75**

**Table 8:** Comparative experimentson the GWO module utilizing across Taiwan Credit Default Data and Home Credit Default Risk Data. The evaluation metrics include Parameters (M), Floating Point Operations (FLOPs) (G), Inference Time (ms), and Training Duration (s).

Model	Taiwan Credit Default Data				Home Credit Default Risk Data			
	P(M)	F(G)	I(ms)	T(s)	P(M)	F(G)	I(ms)	T(s)
Adam	385.12	301.01	293.34	387.02	281.07	257.1	336.7	386.4
Bayesian	382.6	266.39	240.62	292.85	376.74	298.3	217.41	398.9
PSO	307.18	322.61	232.23	289.31	351.24	281.94	385.36	398.31
**Ours 128.86**	**151.37**	**227.02**	**194.6**	**207.98**	**220.13**	**202.53**	**194.93**	

## Conclusion

This study proposes the CNN-BiGRU model for credit score prediction, combining a convolutional neural network (CNN) and a bidirectional gated recurrent unit (BiGRU). The Gray Wolf Optimization (GWO) algorithm optimizes it. Experiments on multiple datasets verify the model’s superior performance.

Although our model has achieved excellent results on multiple datasets, the generalization ability of the model still needs to be further verified, especially when facing different data distributions and unknown scenarios. In addition, we also recognize that the robustness of the model in dealing with noisy data needs to be strengthened. The presence of noisy data may affect the prediction accuracy of the model. Future research will focus on how to optimize data preprocessing and introduce regularization techniques to enhance the stability and adaptability of the model in noisy environments. Secondly, although we have improved the training and inference efficiency of the model, when dealing with larger-scale data, we still need to consider the balance between computing resources and time costs. In order to improve the scalability of the model, we will explore how to optimize the computational efficiency of the model, especially in the inference phase, to ensure that it can provide fast and efficient predictions in real-time applications.

Nevertheless, this paper still successfully built a composite model that integrates CNN and BiGRU, and improved the model performance through the GWO algorithm, providing strong support for deep learning methods in the field of credit scoring. Future research will further expand the scope of application of the model and enhance its robustness and scalability in different financial scenarios. In addition, we will also work on optimizing the real-time performance of the model, exploring how to better integrate it with financial business needs, and providing financial institutions with smarter and more reliable credit assessment tools.
